# Clinical Utility of Venous Blood Gas Analysis for the Evaluation of Psychogenic Hyperventilation in the Emergency Department

**DOI:** 10.7759/cureus.12273

**Published:** 2020-12-25

**Authors:** Seigo Urushidani, Akira Kuriyama, Masami Matsumura

**Affiliations:** 1 Emergency and Critical Care Center, Kurashiki Central Hospital, Kurashiki, JPN; 2 Division of General Medicine, Center for Community Medicine, Jichi Medical University School of Medicine, Shimotsuke, JPN

**Keywords:** hyperventilation, blood gas analysis, respiratory alkalosis, hyperlactatemia

## Abstract

Background

Patients with psychogenic hyperventilation frequently visit emergency departments (EDs). Arterial blood gas (ABG) analysis is performed to evaluate patients with dyspnea. This may show respiratory alkalosis in patients with hyperventilation. ABG may also reveal elevated serum lactate levels, although psychogenic hyperventilation syndrome is a benign condition. However, arterial puncture is a painful and risky procedure. We hypothesized that venous blood gas (VBG) analysis would be sufficient for evaluating patients with suspected psychogenic hyperventilation.

Objectives

To compare the clinical utility of VBG analysis with ABG analysis for evaluating psychogenic hyperventilation.

Methods

This was a single-center retrospective cross-sectional study of patients aged ≥18 years with psychogenic hyperventilation attending a tertiary care hospital. We extracted data on age, sex, vital signs, blood gas components, and serum lactate. Spearman’s rank correlation coefficient (ρ) was used to examine the associations between the serum lactate levels and the carbon dioxide partial pressure (PCO_2_) in the ABG and VBG groups.

Results

A total of 236 patients (ABG group, n=57; VBG group, n=179) were included in the analysis. Both the ABG and VBG groups had respiratory alkalosis and similarly elevated serum lactate levels (p=0.44). The PCO_2 _and serum lactate levels were inversely correlated, and the ρ values were −0.74 and −0.50 for the ABG and VBG groups, respectively (both p<0.001). In addition, the bicarbonate ion ($\textrm{HCO}_{3}^{-}$) level was inversely correlated with the serum lactate level, and the pH was positively correlated with the serum lactate levels in both the ABG and VBG groups.

Conclusions

Among patients with psychogenic hyperventilation, respiratory alkalosis, and the correlation between the PCO_2_ and serum lactate levels were similar in the ABG and VBG groups, indicating that VBG analysis might be used as an alternative to ABG analysis for evaluating psychogenic hyperventilation.

## Introduction

Psychogenic hyperventilation syndrome is a common medical condition. Pfortmueller et al. reported that the incidence of psychogenic hyperventilation among adult patients who presented to an emergency department (ED) was 0.3% [1]. The symptoms of psychogenic hyperventilation include anxiety, dizziness, or paresthesia, and can sometimes mimic other causes of dyspnea. However, the diagnostic criteria for psychogenic hyperventilation have not been clearly defined [2].

Blood gas analysis is used in the evaluation of hyperventilation syndrome. It often reveals respiratory alkalosis in patients with psychogenic hyperventilation, whereas respiratory or metabolic acidosis, with or without respiratory compensation, is often observed in critically ill patients with hyperventilation [3]. Hyperlactatemia is present in both critically ill patients and in patients with psychogenic hyperventilation. Studies of arterial blood gas (ABG) analysis in patients with psychogenic hyperventilation have shown an inverse correlation between the arterial partial pressure of carbon dioxide (PaCO_2_) and the serum lactate level [2,4].

Despite the potential utility of ABG analysis for the evaluation of patients with suspected psychogenic hyperventilation, the required arterial puncture is painful and confers a risk of complications such as hematoma or peripheral nerve injury [5-7]. Furthermore, patients with psychogenic hyperventilation are not in a hypoxic state, and pulse oximetry should be sufficient to monitor oxygen saturation in the peripheral arteries (SpO_2_). Therefore, although ABG analysis is useful, it may not be necessary for the evaluation of psychogenic hyperventilation, and a noninvasive alternative method may be preferable.

This study was undertaken to evaluate whether respiratory alkalosis and hyperlactatemia, and an inverse correlation between the serum lactate level and partial pressure of carbon dioxide (PCO_2_), could be detected in both the arterial and venous blood of patients with suspected psychogenic hyperventilation. This study aimed to determine whether venous blood gas (VBG) could be used as a substitute for ABG when evaluating patients with suspected psychogenic hyperventilation who present to the ED.

## Materials and methods

Study design and setting

This single-center, retrospective, cross-sectional study was conducted from January 2015 to December 2018 at the ED of Kurashiki Central Hospital, a 1,166-bed tertiary care center in Okayama, Japan. The ED at the study center treats approximately 70,000 emergency patients annually, including nearly 10,000 patients who are transferred by ambulance or helicopter. In the ED, investigations such as blood examinations or radiography are undertaken at the discretion of the treating and/or attending physicians. We ascertained the final diagnoses of patients from the ED discharge notes in the electronic medical records. The study protocol was approved by the institutional ethics committee of Kurashiki Central Hospital (approval no. 2739) and was conducted in accordance with the guidelines for research on human participants in Japan. The requirement for informed consent was waived because of the retrospective study design.

Selection of participants

We searched the hospital’s electronic medical records and extracted data on patients who were diagnosed with psychogenic hyperventilation by the treating physician. Adult patients (age ≥18 years) who were diagnosed with psychogenic hyperventilation at the time of discharge from the ED and for whom ABG or VBG data were available were included in this study. For the purposes of this study, we defined psychogenic hyperventilation as: (1) an episode of an excessive number or depth of respiration noted by the patient, an accompanying person, or emergency medical service staff; and (2) an improvement in the patient’s status without specific treatment with an uneventful discharge from the ED. Two authors (SU and AK) independently reviewed the medical records of all the patients identified by the search, and excluded patients with the following conditions: (1) SpO_2_ <96% in room air, (2) initiation of supplemental oxygen administration before arrival at the ED, (3) a suspected organic or somatic cause of hyperventilation based on the history, vital signs, electrocardiography, chest radiography, or blood test results, and (4) hospital admission after an evaluation in the ED. We did not conduct the hyperventilation provocation test on all patients, because it was not routine to apply the hyperventilation provocation test for spontaneously improved patients with psychogenic hyperventilation in Japan.

Measurements

Arterial blood samples were drawn from the patient’s radial or femoral artery, and venous blood samples were obtained from any peripheral vein. The blood samples were drawn into a heparinized plastic syringe and immediately subjected to a blood gas analysis using an ABL 800 FLEX (Radiometer Medical K.K., Tokyo, Japan) in the ED. The test results were automatically transferred and recorded in patients’ electronic medical records.

For each patient, we extracted data on the age, sex, vital signs, pH, partial pressure of oxygen (PO_2_), PCO_2_, bicarbonate ion ($\textrm{HCO}_{3}^{-}$), sodium ion (Na^+^), potassium ion (K^+^), chloride ion (Cl^−^), ionized calcium ion (Ca^2+^), and lactate for the statistical analysis.

Statistical analysis

As the continuous variables were non-normally distributed, they were analyzed using the non-parametric Mann-Whitney U-test. The results were reported as medians and interquartile ranges (IQRs). Variables with dichotomous outcomes (e.g., sex) were evaluated using Fisher’s exact test. As the previous study had shown that the correlation between PCO_2_ and lactate was not linear, Spearman’s rank correlation coefficient (ρ) was calculated to assess the associations between serum lactate level and pH, PCO_2_, or $\textrm{HCO}_{3}^{-}$ in each study group (ABG and VBG analysis) [2,4]. Using the same procedure, we conducted a sensitivity analysis using data from the included patients with psychogenic hyperventilation who had a respiratory rate $\geq$22 breaths/min in the ED. The statistical analyses were performed using EZR version 1.37 [8].

## Results

Characteristics of study participants

**Figure 1:**
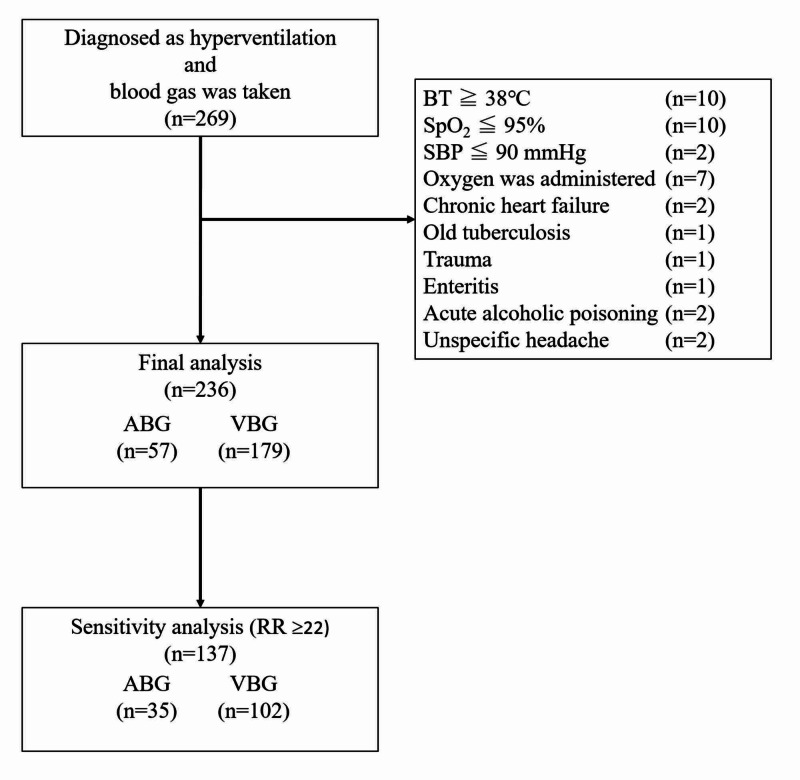
Selection of Study Participants Abbreviations: ED: emergency department, BT: body temperature, SpO_2_: percutaneous oxygen saturation, SBP: systolic blood pressure, ABG: arterial blood gas, VBG: venous blood gas.

Two hundred sixty-nine patients were diagnosed with hyperventilation and underwent a blood gas analysis during the study period. Of these 269 patients, 33 patients were excluded based on the exclusion criteria and 236 patients (median age: 48 years, IQR: 32-69 years; median respiratory rate on arrival at the ED: 24 breaths/minute, IQR: 18-30 breaths/minute; 73.7% female) were included in the final analysis (Figure 1). Table 1 presents the characteristics of the study participants. Of the 236 patients, 57 and 179 were evaluated using ABG analysis and VBG analysis, respectively. No patients underwent both ABG and VBG analysis. The median age was lower in the VBG group than in the ABG group, while the sex distribution and the median respiratory rate were similar in both groups.

**Table 1:** Characteristics and Blood Gas Analyses of Study Participants The data are presented as median with interquartile range. *Fisher’s exact test. ^†^Mann-Whitney U test. Abbreviations: PO_2_: partial pressure of oxygen, PCO_2_: partial pressure of carbon dioxide, $\textrm{HCO}_{3}^{-}$: bicarbonate ion; K, potassium ion.

	Arterial	Venous	p-Value
Number	57	179	
Female (%)	67	76	0.171*
Age (years)	65 (38-77)	45 (31-65)	<0.001^†^
Respiratory rate	24 (20-30)	24 (18-30)	0.90^†^
pH	7.61 (7.52-7.65)	7.52 (7.44-7.58)	<0.001^†^
PO_2_ (mmHg)	104 (91-121)	36 (29-46)	<0.001^†^
PCO_2_ (mmHg)	20.5 (17.4-25.8)	28.3 (23.3-35.7)	<0.001^†^
$\textrm{HCO}_{3}^{-}$ (mmol/L)	20.4 (19.3-22.7)	23.5 (21.8-25.0)	<0.001^†^
Lactate (mmol/L)	2.6 (1.5-3.4)	2.4 (1.8-3.5)	0.44^†^
K (mEq/L)	3.3 (3.1-3.6)	3.5 (3.2-3.7)	0.003^†^

Main results

**Figure 2:**
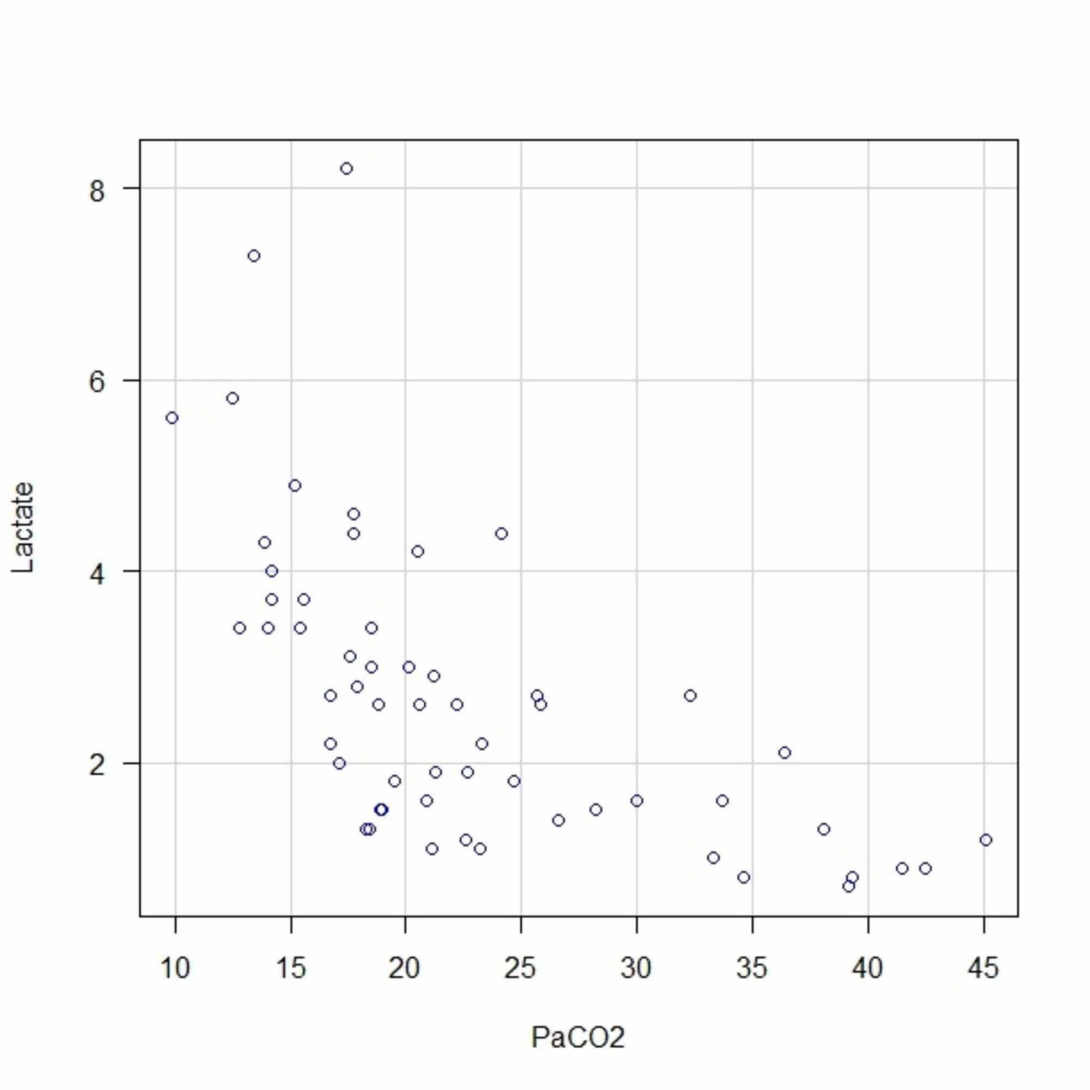
Scatter Plot of Partial Pressure of Carbon Dioxide and Serum Lactate Levels in Arterial Blood Abbreviations: PaCO_2_: partial pressure of carbon dioxide in arterial blood.

**Figure 3:**
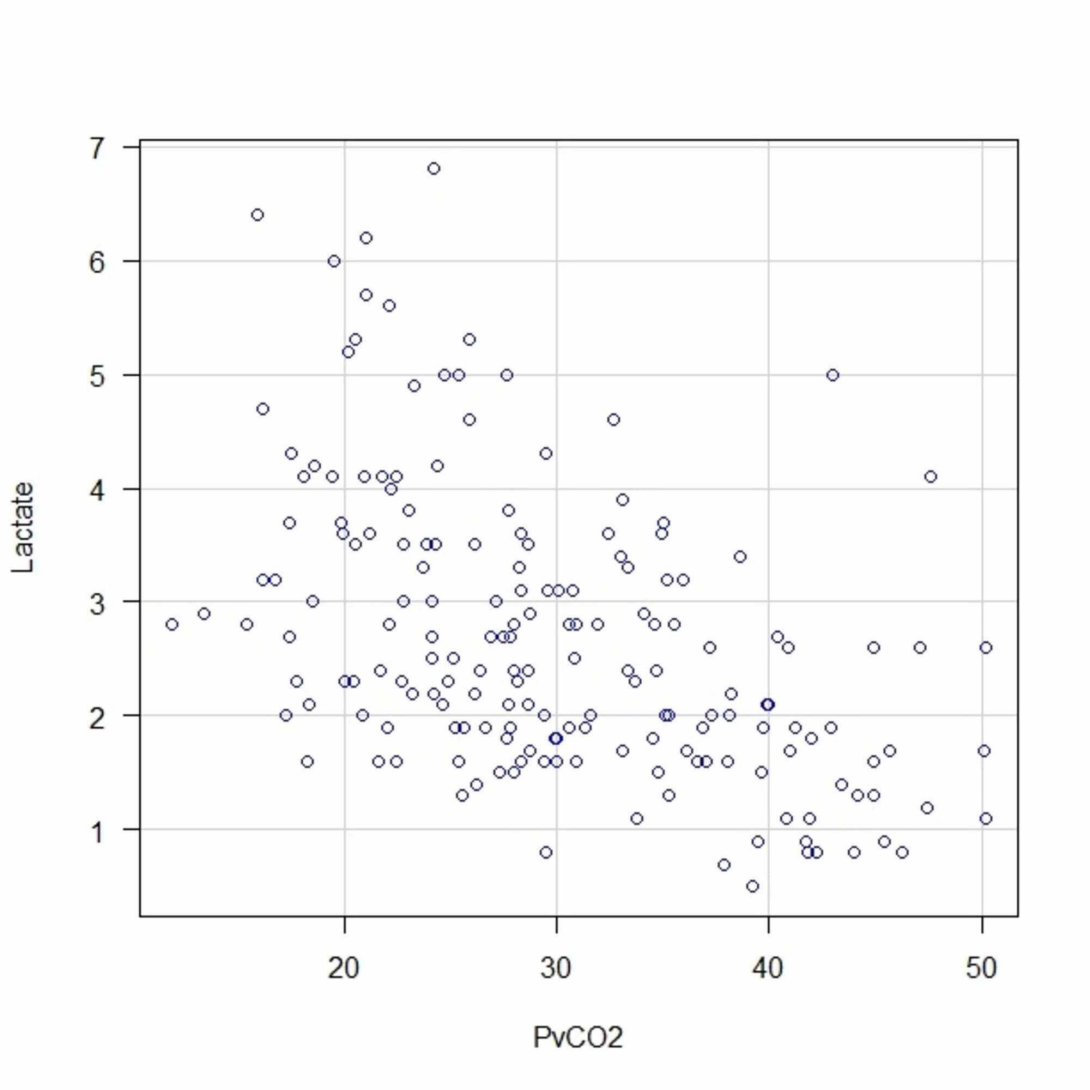
Scatter Plot of Partial Pressure of Carbon Dioxide and Serum Lactate Levels in Venous Blood Abbreviations: PvCO2: partial pressure of carbon dioxide in venous blood.

Although the degree of alkalemia and hypocapnia differed between the study groups, both the ABG and VBG groups had respiratory alkalosis and elevated serum lactate levels (p=0.44). The Spearman’s rank correlation coefficients (ρ) for the association between the PCO_2 _and serum lactate level were −0.74 and −0.50 in the ABG and VBG groups, respectively. Furthermore, in both groups, the $\textrm{HCO}_{3}^{-}$ (mmol/L)level was inversely correlated with the serum lactate levels, and the pH was positively correlated with the serum lactate levels (Table 2). The correlations between the PCO_2_ and serum lactate levels in the ABG and VBG groups are shown as scatterplots in Figures 2 and 3, respectively.

**Table 2:** Spearman’s Rank Correlation Coefficient Between Serum Lactate Levels and pH, PCO2 and HCO3- Abbreviations: PCO_2_: partial pressure of carbon dioxide, HCO3-: bicarbonate ion.

	Arterial		Venous	
	ρ	p	ρ	p
Lactate/pH	0.56	<0.001	0.42	<0.001
Lactate/PCO_2_	−0.74	<0.001	−0.50	<0.001
Lactate/$\textrm{HCO}_{3}^{-}$	−0.74	<0.001	−0.51	<0.001

​​​​​​Sensitivity analysis

A total of 137 patients with a respiratory rate $\geq$22/min, 35 in the ABG group and 102 in the VBG group, were included in the sensitivity analysis (Figure 1). Their characteristics are shown in Table 3. Patients in both groups had respiratory alkalosis and hyperlactatemia, with an inverse correlation between the PCO_2_ and serum lactate levels (Table 4).

**Table 3:** Characteristics and Blood Gas Analyses of Study Participants (RR ≥22) The data are presented as median with interquartile range. *Fisher’s exact test. ^†^Mann-Whitney U test. Abbreviations: PO_2_: partial pressure of oxygen, PCO_2_: partial pressure of carbon dioxide, HCO_3_^-^: bicarbonate ion, K: potassium ion.

	Arterial	Venous	p-value
Number	35	102	
Woman (%)	66	80	0.104*
Age (years)	63 (38-77)	47 (32-69)	0.046^†^
Respiratory rate	28 (24-30)	30 (24-34)	0.332^†^
pH	7.63 (7.55-7.66)	7.53 (7.45-7.59)	<0.001^†^
PO_2_ (mmHg)	104 (92-121)	36 (29-45)	<0.001^†^
PCO_2_ (mmHg)	19.5 (17.7-23.7)	28.1 (21.3-34.8)	<0.001^†^
HCO_3_^-^ (mmol/L)	20.4 (18.6-22.3)	23.4 (21.4-24.8)	<0.001^†^
Lactate (mmol/L)	2.7 (1.6-3.6)	2.7 (2.0-3.6)	0.47^†^
K (mEq/L)	3.3 (3.1-3.6)	3.5 (3.2-3.7)	0.022^†^

**Table 4:** Spearman’s Rank Correlation Coefficient Between Serum Lactate Levels and pH, PCO2 and HCO3- (RR ≥22) Abbreviations: PCO_2_: partial pressure of carbon dioxide, HCO_3_^-^: bicarbonate ion.

	Arterial		Venous	
	ρ	p	ρ	p
Lactate/pH	0.31	0.073	0.34	<0.001
Lactate/PCO_2_	−0.58	<0.001	−0.45	<0.001
Lactate/HCO_3_^-^	−0.71	<0.001	−0.54	<0.001

## Discussion

Patients in both the ABG and VBG groups had respiratory alkalosis, elevated serum lactate levels, and an inverse correlation between the serum lactate and PCO_2_ levels. Elevated serum lactate levels are often observed in ED patients and are usually attributed to shock caused by trauma or sepsis [9,10]. Lactic acidosis is generally caused by tissue hypoxia or anaerobic metabolism and is associated with poor outcomes in some clinical conditions [11]. Conversely, in psychogenic hyperventilation, CO_2_ is lost during expiration, with a resultant reduction in the PCO_2_ level. As CO_2_ is a weak acid, the decreased PCO_2_ level leads to an increased pH (alkalosis) [2,12]. Subsequently, alkalosis activates phosphofructokinase, a rate-limiting enzyme within the glycolytic pathway, and accelerates glycolysis, which leads to an increase in the serum lactate level [12-14]. Lactate is mainly metabolized in the liver. However, hepatic lactate metabolism is impaired and the lactate half-life extended when the intracellular pH is outside the range 7.05-7.30. This may explain the elevated serum lactate levels in patients with psychogenic hyperventilation [14].

Previous studies have demonstrated elevated serum lactate levels in arterial blood of patients with psychogenic hyperventilation [2,4]. In this study, similarly elevated serum lactate levels were present in venous blood of patients with psychogenic hyperventilation. In this study, there were differences between the ABG and VBG groups in the pH, and the PCO_2_ and $\textrm{HCO}_{3}^{-}$ levels, but not the serum lactate level. These differences were similar to those reported in a previous meta-analysis of the extent of agreement between arterial and venous samples for blood gas analysis [15]. Although the increases in the PCO_2_ and $\textrm{HCO}_{3}^{-}$ levels were smaller in the ABG group than in the VBG group, a similar level of respiratory alkalosis and an inverse correlation between the PCO_2_ and serum lactate levels were present in both groups. In the sensitivity analysis, the positive correlation between the blood pH and the serum lactate level in the ABG group was not statistically significant, although the point estimate was similar to that of the primary analysis. The lack of a statistically significant difference might be due to the small sample size of the ABG group in the sensitivity analysis.

Spearman’s rank correlation coefficient between PaCO_2_ and lactate in this study (ρ=−0.74, p<0.01) was greater than that reported in a previous study by ter Avest et al. (ρ=−0.50, p<0.01) [2]. According to previous reports, hyperlactatemia is more likely to occur in hypocapnic than in normocapnic patients [2,4]. The median PaCO_2_ in the study population was 20.5 mmHg, which was less than that reported by ter Avest et al. (32.3 mmHg). Accordingly, the participants may have had higher levels of respiratory alkalosis, and subsequently, higher serum lactate levels [2].

To the best of our knowledge, this study is the first to demonstrate that respiratory alkalosis and elevated serum lactate can be detected in venous blood, and that the PCO_2_ is inversely correlated with serum lactate levels in the venous blood of patients with psychogenic hyperventilation. Our findings suggest that VBG may be used as a substitute for ABG in the investigation of patients with suspected psychogenic hyperventilation.

This study had some limitations. First, there are no established or validated diagnostic criteria for psychogenic hyperventilation. We detected respiratory alkalosis and an inverse correlation between PCO_2_ and serum lactate levels in both the ABG and VBG groups in the primary and sensitivity analyses. These findings suggest that the blood gas analysis can reveal respiratory alkalosis and an inverse correlation between the PCO_2_ and serum lactate levels in patients with suspected psychogenic hyperventilation, even if tachypnea has improved by the time of arrival at the ED. Thus, it may be appropriate to use venous blood for blood gas analysis of patients with suspected psychogenic hyperventilation in ED practice among patients who meet the inclusion criteria that we used in this study. Second, because this was a retrospective study, we were unable to collect data from arterial and venous blood samples drawn concurrently from the same patients. However, conducting such a study prospectively would require patients to experience blood sampling pain and discomfort twice, and this might not be ethically acceptable in patients with suspected psychogenic hyperventilation. Thus, our study design may be more feasible. Third, to verify the utility of VBG analysis in the patients with psychogenic hyperventilation alone, we excluded the patients whose SpO_2_ was <96% in room air and patients who had been administered oxygen before arrival at the ED. Eight patients with psychogenic hyperventilation (four patients in the ABG group and four patients in the VBG group) had been administered oxygen before arrival, and were thus excluded from the main analysis. However, respiratory alkalosis, hypocapnia, and elevated serum lactate levels were similarly detected in both the ABG and VBG groups when these eight patients were included. Fourth, this study investigated arterial and venous blood gas only in patients with psychogenic hyperventilation to evaluate their condition. Future studies are warranted to investigate whether blood gas analysis can distinguish psychogenic hyperventilation from other critical conditions.

## Conclusions

In conclusion, respiratory alkalosis, hypocapnia, and elevated serum lactate levels may be detectable in the venous blood samples of patients with psychogenic hyperventilation. The correlation between the PCO_2_ and serum lactate levels was similar in the VBG and ABG groups. Therefore, VBG analysis might be a suitable substitute for ABG analysis when evaluating patients with suspected psychogenic hyperventilation.

## References

[1] Pfortmueller CA, Pauchard-Neuwerth SE, Leichtle AB, Fiedler GM, Exadaktylos AK, Lindner G (2015). Primary hyperventilation in the emergency department: a first overview. PLoS One.

[2] ter Avest E, Patist FM, Ter Maaten JC, Nijsten MW (2011). Elevated lactate during psychogenic hyperventilation. Emerg Med J.

[3] Mizock BA, Falk JL (1992). Lactic acidosis in critical illness. Crit Care Med.

[4] Ueda Y, Aizawa M, Takahashi A, Fujii M, Isaka Y (2009). Exaggerated compensatory response to acute respiratory alkalosis in panic disorder is induced by increased lactic acid production. Nephrol Dial Transplant.

[5] Neviaser RJ, Adams JP, May GI (1976). Complications of arterial puncture in anticoagulated patients. J Bone Joint Surg Am.

[6] Okeson GC, Wulbrecht PH (1998). The safety of brachial artery puncture for arterial blood sampling. Chest.

[7] Stroud S, Rodoribuez R (2004). Arterial puncture and cannulation. Emergency Medicine Procedure.

[8] Kanda Y (2013). Investigation of the freely available easy-to-use software ‘EZR’ for medical statistics. Bone Marrow Transplant.

[9] Mikkelsen ME, Miltiades AN, Gaieski DF (2009). Serum lactate is associated with mortality in severe sepsis independent of organ failure and shock. Crit Care Med.

[10] Rishu AH, Khan R, Al-Dorzi HM, Tamim HM, Al-Qahtani S, Al-Ghamdi G, Arabi YM (2013). Even mild hyperlactatemia is associated with increased mortality in critically ill patients. Crit Care.

[11] Kraut JA, Madias NE (2014). Lactic acidosis. N Engl J Med.

[12] Hood VL, Tannen RL (1983). pH control of lactic acid and keto acid production: a mechanism of acid-base regulation. Miner Electrolyte Metab.

[13] Suarez N, Conway N, Pickett T (2013). Panic-related hyperventilation resulting in hypophosphataemia and a high lactate. BMJ Case Rep.

[14] Druml W, Grimm G, Laggner AN, Lenz K, Schneeweiss B (1991). Lactic acid kinetics in respiratory alkalosis. Crit Care Med.

[15] Bloom BM, Grundlingh J, Bestwick JP, Harris T (2014). The role of venous blood gas in the emergency department: a systematic review and meta-analysis. Eur J Emerg Med.

